# Overexpression of GNPDA1 in head and neck squamous cell carcinoma: Prognostic significance, immune infiltration, and correlation with cancer cell immune evasion

**DOI:** 10.1097/MD.0000000000042561

**Published:** 2025-05-23

**Authors:** Peng Liu, Dunhui Yang, Ruixia Ma

**Affiliations:** a Second Clinical Medical College, Ningxia Medical University, Yinchuan, Ningxia, PR China; b Otolaryngology Department, The First People’s Hospital of Yinchuan, Otolaryngology Head and Neck Surgery Hospital, Yinchuan, Ningxia, PR China; c Department of Graduate and Scientific Research, Zunyi Medical University Zhuhai Campus, Zhuhai, PR China.

**Keywords:** glucosamine-6-phosphate isomerase 1, head and neck squamous cell carcinoma, immune evasion, immune infiltration, prognostic biomarker

## Abstract

To evaluate the correlation between glucosamine-6-phosphate isomerase 1 (GNPDA1) expression and prognosis, immune infiltration, and immune evasion in head and neck squamous cell carcinoma (HNSCC). We analyzed the expression of GNPDA1 in HNSCC tissues and obtained RNA sequence data from the Cancer Genome Atlas (TCGA) database. Kaplan–Meier survival analysis evaluated the relationship between GNPDA1 expression and advanced tumor stage, TNM stage, grading, and gender. Co-expressed genes with GNPDA1 were identified using TCGA data and annotated through gene ontology and Kyoto encyclopedia of genes and genomes analyses. A protein–protein interaction network was constructed using the STRING database. Single-sample gene set enrichment analysis was conducted based on TCGA and TIMER 2.0 databases to assess the correlation between GNPDA1 and immune infiltration. In addition, the location of GNPDA1 in tumor cell and immune cell structures was identified by the tumor immune stromal cells helper database, and potential protein-interacting molecules of GNPDA1 were elucidated in the STRING database. Potential GNPDA1 gene functions were assessed using gene set enrichment analysis. Our results indicate that the expression of GNPDA1 is elevated in HNSCC tissues (*P* < .05). GNPDA1 expression was positively correlated with tumor malignancy (*P* < .05) and negatively correlated with patient prognosis (*P* < .05). There was a significant correlation between high expression of GNPDA1 and advanced tumor stage, N-stage, or G-grade, and it was associated with gender. High GNPDA1 expression was associated with increased infiltration of resting CD4^+^T cells, macrophages M1 and M2, resting natural killer cells, monocytes, eosinophils, and naïve B cells (*P* < .05). In contrast, low GNPDA1 expression was associated with increased infiltration of activated natural killer cells, neutrophils, activated mast cells, macrophages M0, plasma cells, activated dendritic cells, CD8^+^T cells, memory B cells, regulatory T cells (Tregs), and naïve CD4^+^T cells (*P* < .05). In addition, GNPDA1 was observed to be closely associated with various immune evasion-related genes in HNSCC. The results of this study suggest that GNPDA1 can serve as a potential prognostic marker and therapeutic target for HNSCC and may be a key gene mediating immune evasion in HNSCC.

## 1. Introduction

Head and neck cancer is a prevalent form of cancer globally, with head and neck squamous cell carcinoma (HNSCC) accounting for up to 90% of cases.^[1]^ This cancer typically originates in the larynx, pharynx, and mouth.^[2]^ Statistics from 2018 indicate that over 8,90,000 cases of HNSCC were diagnosed worldwide, resulting in approximately 4,50,000 deaths and a mortality rate of 50%.^[3]^ Several factors have been identified as contributing to the development of HNSCC, including long-term smoking, heavy alcohol consumption, and human papillomavirus infection.^[4]^ Currently, the treatment of HNSCC primarily involves a combination of surgery, radiation therapy, and chemotherapy.^[5]^ With increasing research on the tumor microenvironment (TME), immunotherapy has emerged as a promising treatment option for HNSCC. Pembrolizumab, a PD-1/PD-L1 inhibitor, has been approved as treatment for HNSCC due to its ability to significantly improve overall survival (OS) in patients.^[5–7]^ However, many patients still do not respond to pembrolizumab,^[8]^ and the 5-year survival rate for HNSCC remains below 50%.^[9]^ Given these challenges, it is imperative to delve deeper into the intricacies of the TME and identify new therapeutic targets for immunotherapy. By understanding the diverse mechanisms underlying HNSCC progression, researchers can develop more effective treatments and improve patient outcomes.

Glucosamine-6-phosphate isomerase 1 (GNPDA1) belongs to the glucosamine-6-phosphate deaminase family and serves as a key enzyme linking hexosamine metabolism to the glycolytic pathway.^[10]^ It facilitates the breakdown of hexosamine derived from glycolipids, glycoproteins, and sialic acid into phosphate sugars, which serve as an energy source for tumor cells.^[11]^ High expression of GNPDA1 has been observed in various cancers, including liver cancer,^[11]^ pancreatic cancer^[12]^ and colorectal cancer. Furthermore, its upregulation is significantly associated with advanced tumor stages, TNM staging, higher grades, and poor prognosis in liver cancer patients. Experiments have demonstrated that GNPDA1 promotes proliferation, migration, invasion, and inhibits apoptosis of HCC cells.^[11]^ Additionally, studies on colorectal cancer cells treated with caffeic acid phenyl ester have shown that GNPDA1 expression is upregulated along with glutathione peroxidase 1, while proteasome subunit alpha 1 and phosphoribosyl aminotransferase 1 are downregulated. These findings further support the notion that high GNPDA1 expression is linked to poor tumor prognosis.^[13]^ Despite these advancements, current research on the association between GNPDA1 expression and cancer is relatively limited, and no studies have reported the correlation between GNPDA1 and HNSCC. Therefore, this study aimed to investigate the impact of GNPDA1 on HNSCC progression and prognosis, providing a theoretical basis for identifying new therapeutic targets for HNSCC treatment. By examining the role of GNPDA1 in HNSCC, this research seeks to offer insights into potential therapeutic strategies that could improve patient outcomes.

## 2. Materials and methods

### 2.1. GNPDA1 expression and localization in cancer varied

To assess GNPDA1 expression in 23 different tumors, we utilized the TIMER 2.0 database.^[14]^ RNA sequence data and clinical information related to HNSCC were analyzed using the Cancer Genome Atlas (TCGA) database. Using the “limma” R software package, we visualized the differential expression of GNPDA1 between tumor tissues and adjacent non-tumor tissues. For data visualization, we employed the “ggplot2” and “ggpubr” R software packages.

### 2.2. Single-cell analysis of GNPDA1 in HNSCC

Using the Immuno-Oncology Single Cell Center website (https://tisch.comp-genomics.org/),^[15]^ we analyzed GNPDA1 expression across major cell lineages. A heatmap displaying GNPDA1 expression in 16 single-cell types was generated. Additionally, we examined GNPDA1 expression levels in various cell clusters within the HNSC_GSE103322 dataset.

### 2.3. Survival analyze

Patient data from the TCGA database with clinical HNSCC was utilized to evaluate the impact of GNPDA1 on OS using Kaplan–Meier (K–M) survival analysis. For visualization, the “survminer” R software packages were employed. Based on a 50% criterion, patients were stratified into high and low GNPDA1 expression groups.

### 2.4. GNPDA1-clinical data correlation

To further explore GNPDA1’s clinical significance, we analyzed its expression and clinical data using the “ggplot2” R package.

### 2.5. *GNPDA1 protein–protein interactions, differential expression genes and gene fusion analysis between high and low expression of GNPDA1*

To investigate the protein–protein interactions involving GNPDA1 in HNSCC, we conducted a correlation analysis using TCGA data and calculated the Pearson coefficient. We selected genes with a Pearson coefficient > 0.6 and *P* < .001 for further analysis and plotted a donut chart using the “ggplot2” R package, highlighting the top 12 genes co-expressed with GNPDA1. For median expression level of GNPDA1, we divide HNSCC patients into high expression and low expression. To identify differentially expressed genes (DEGs) between these 2 groups, we used the “limma” R package for visualization.^[16]^ Significant differences were determined using a threshold of logFC > 0.5 and *P* < .05. We displayed the first 50 significantly upregulated and downregulated genes using a heatmap generated by the “pheatmap” R package. Furthermore, we performed gene ontology (GO) and Kyoto encyclopedia of genes and genomes analyses on the DEGs using the “clusterProfiler” and “org.Hs.e.g.db” R packages. These analyses provided insights into the biological processes, molecular functions, cellular components, and pathways associated with GNPDA1 expression in HNSCC.^[17,18]^

### 2.6. Immunotherapy, TME, TMB, and checkpoint gene analysis

Utilizing gene expression data, Cell type Identification By Estimating Relative Subsets Of Transcriptional profiles was employed to determine the infiltration levels of various immune cells in tumor samples. This method allowed for the estimation of 22 different immune cell types present within the organization. The relationship between the immune cell counts and the expression of GNPDA1 was analyzed using the Spearman method, with statistical significance set at *P* < .05. Visual representations of the data were generated using the “ggplot” R software package to create block plots and scatter plots. Subsequently, a correlation analysis between GNPDA1 and immune checkpoint (ICP) genes was conducted, with statistical significance set at *P* < .001. The results were further visualized using the “corrplot” R software packages. Furthermore, the impact of GNPDA1 on pan-cancer tumor mutation burden (TMB) was explored by evaluating the TMB file downloaded from the University of California, Santa Cruz Xena database, with visualization done through scatter plots^[19]^ (https://xena.ucsc.edu/).

### 2.7. Drug sensitivity

To investigate whether GNPDA1 expression can be used to assess the drug sensitivity of HNSCC patients, a drug sensitivity analysis was conducted.^[20]^ All analyses and results were visualized using the “limma,” “pRRophetic,” “ggplot2” and “ggpubr,” R packages.

### 2.8. Statistical analysis

The relationship between GNPDA1 and clinical outcomes was investigated by analyzing survival curves through a Kaplan–Meier plotter and comparing them with the log-rank test. Additionally, the correlation between GNPDA1 expression and other genes was examined using the Pearson chi-square test. The findings shed light on the potential implications of GNPDA1 in clinical settings.

## 3. Results

### 3.1. Differences in GNPDA1 expression levels between healthy and cancerous tissues vary among different types of cancer

**Figure 1.**
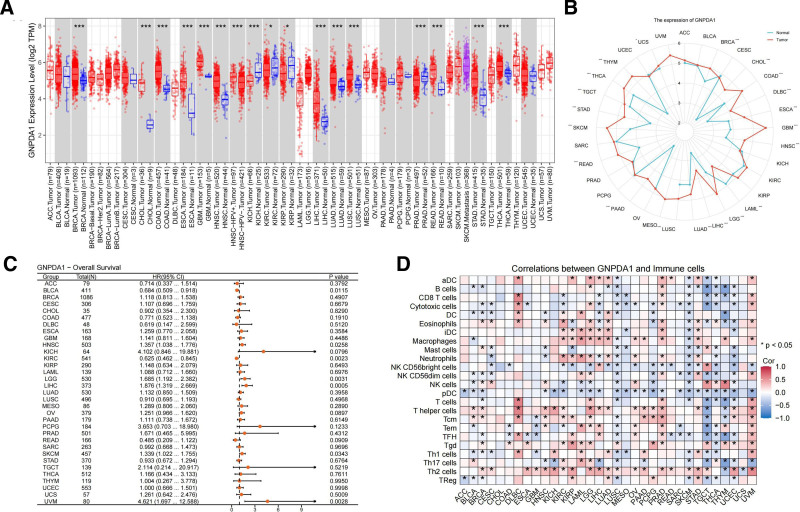
(A) The level of GNPDA1 in 23 cases of malignant tumors in TIMER 2.0 database. (B) Analysis of GNPDA1 levels in 23 cases of malignant tumors using TCGA combined with GTEx database. (C) The relationship between GNPDA1 and overall survival rate of 23 cases of malignant tumors in TCGA database. (D) The relationship between GNPDA1 expression and immune cells in malignant tumors. (*P < .05; ***P < .001.) GNPDA1 = glucosamine-6-phosphate isomerase 1, TCGA = the Cancer Genome Atlas.

**Figure 2.**
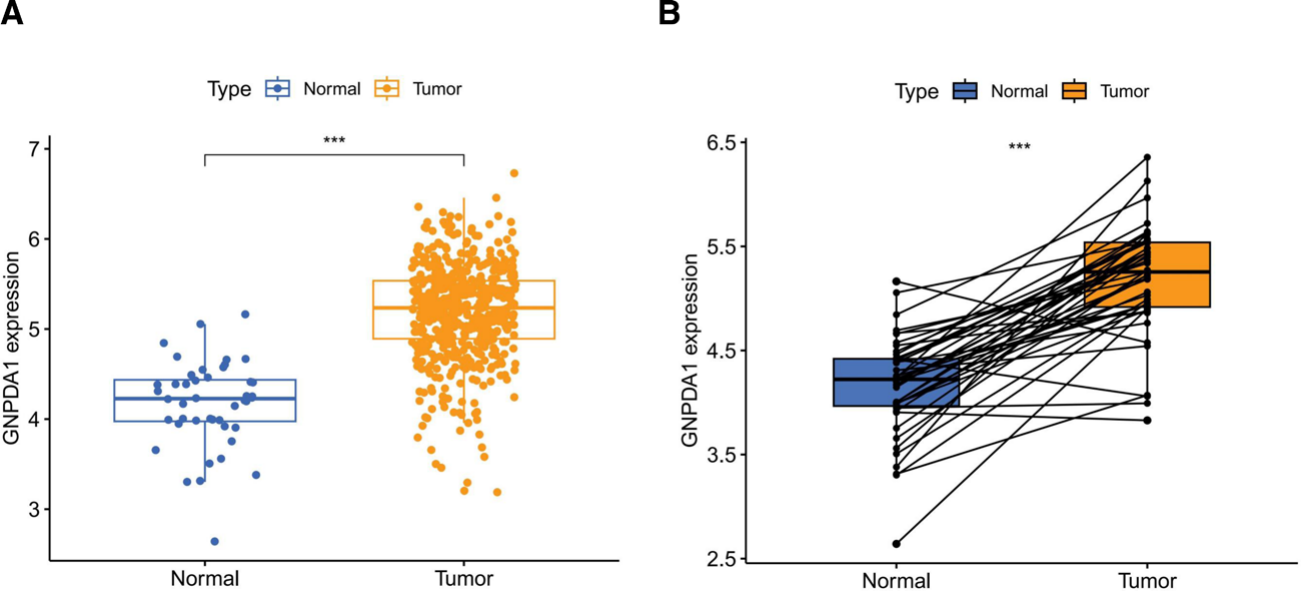
(A) The overall expression level of GNPDA1 in tumor and normal tissues of HNSCC patients. (B) The expression of GNPDA1 in tumor and normal tissues of HNSCC patients. (***P < .001. ) GNPDA1 = glucosamine-6-phosphate isomerase 1, HNSCC = head and neck squamous cell carcinoma.

The analysis of GNPDA1 expression in various types of cancer revealed significant upregulation in 17 out of 23 malignancies compared to normal tissues. The tumors showing increased GNPDA1 levels included BRCA, COAD, HNSC, THCA, CHOL, KICH, GBM, KIRP, READ, KIRC, ESCA, LUSC, PRAD, LUAD, LIHC, and STAD. On the other hand, GNPDA1 was found to be down-regulated in CESC, BLCA, PAAD, PCPG, SKCM, and UCEC (Fig. 1A). These findings were consistent when analyzed using the TCGA combined with GTEx database (Fig. 1B). Moreover, the study examined the OS rates of patients with GNPDA1 expression in the 23 types of cancer under investigation (Fig. 1C). The analysis revealed intriguing relationships between GNPDA1 and immune cells in different tumors. RNA-seq data from the TCGA database were utilized to explore these connections through the single sample gene set enrichment analysis algorithm (Fig. 1D). In addition, the expression levels of GNPDA1 were compared between tumor tissue and normal tissue, as well as their respective paired normal tissues (Fig. 2A, B). These comparisons provided further insights into the role of GNPDA1 in cancer development and its potential as a biomarker for diagnosis or treatment.

### 3.2. GNPDA1 plays a critical role in cancer cell and immune cell functions due to its widespread expression

**Figure 3.**
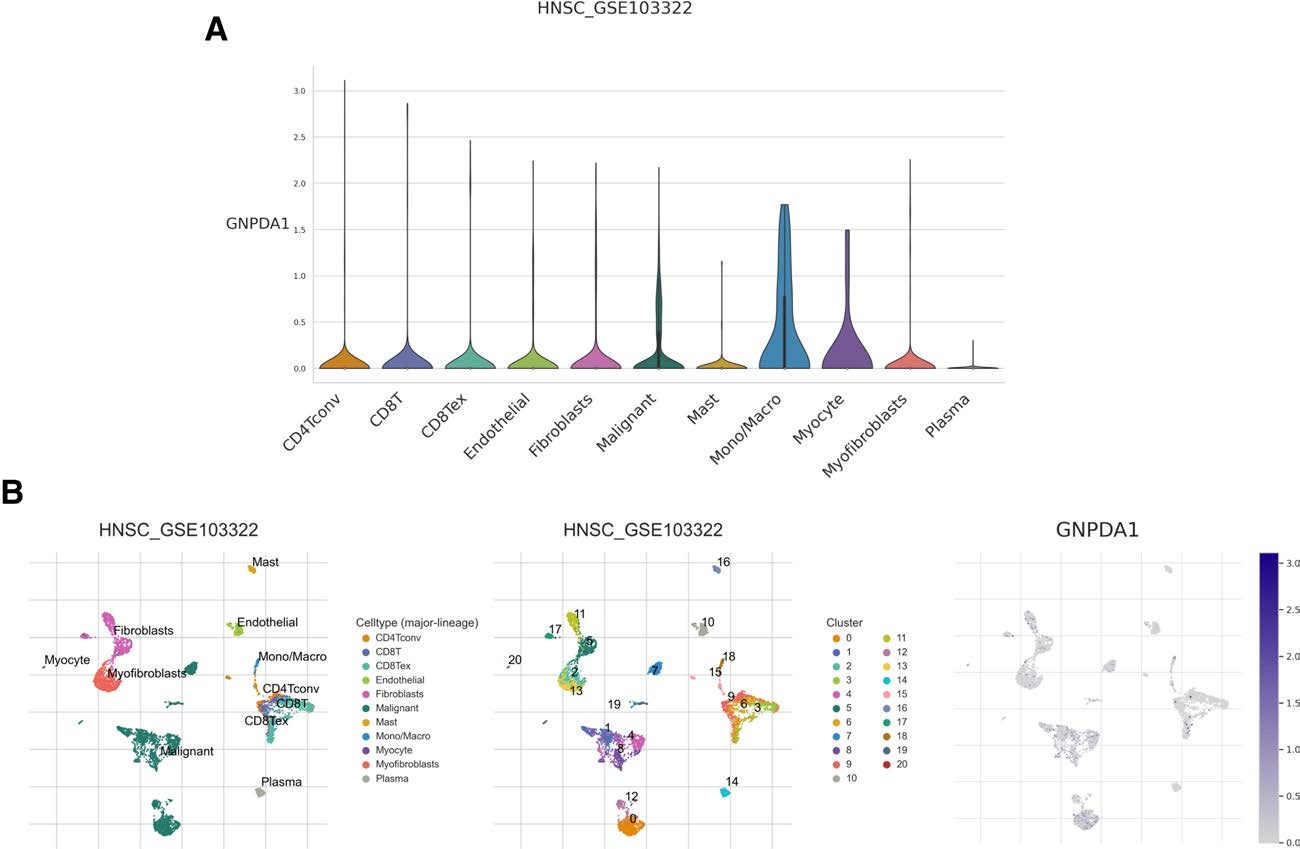
(A) Single-cell sequencing analysis of HNSCC. Single-cell expression of GNPDA1 in HNSCC. (B) Major distribution of GNPDA1 on cell types in the HNSC_GSE103322 dataset. GNPDA1 = glucosamine-6-phosphate isomerase 1, HNSCC = head and neck squamous cell carcinoma.

Analysis of single-cell sequencing data from the TICH database revealed high expression of GNPDA1 in tumor cells and immune cells (Fig. 3A). Specifically, GNPDA1 was found to be highly expressed in T cells and natural killer (NK) cells (Fig. 3B). This suggests a potential role for GNPDA1 in the immune response to tumors, highlighting its importance in tumor cells and immune cells.

### 3.3. High GNPDA1 expression in HNSCC patients is associated with a worse prognosis, indicating correlation with advanced tumor staging

**Figure 4.**
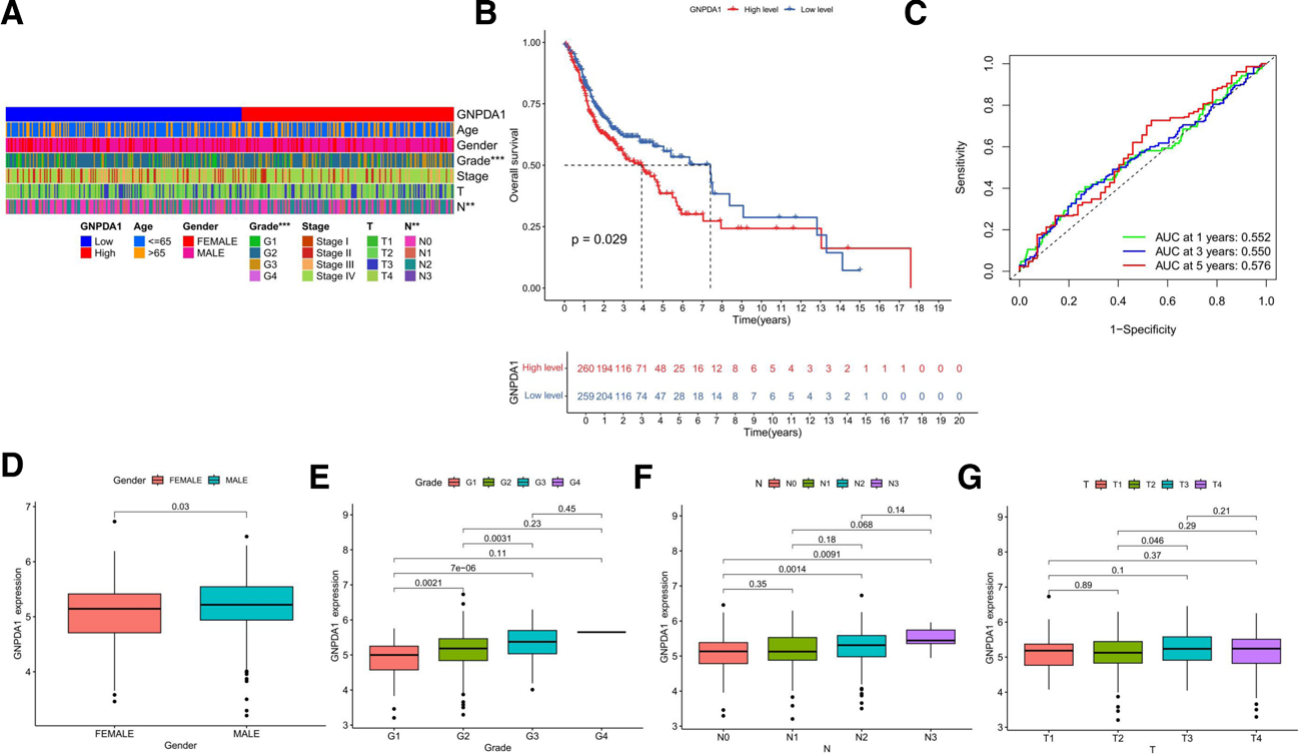
Correlation of GNPDA1 expression with HNSCC prognosis and clinicopathologic data. (A) Correlation of clinicopathologic factors with GNPDA1 expression. (B) Kaplan–Meier curves showing the difference in OS between HNSCC patients with low and high GNPDA1 expression. (C) GNPDA1 predicts the prognosis of patients with 1, 3, and 5-year survival. (D) Expression of GNPDA1 in HNSCC tissues of different genders. (E) Expression of GNPDA1 in HNSCC tissues of different G-stages. (F) Expression of GNPDA1 in HNSCC tissues of different N stages. (G) Expression of GNPDA1 in T-stage HNSCC tissues. (*P < .05.) GNPDA1 = glucosamine-6-phosphate isomerase 1, HNSCC = head and neck squamous cell carcinoma, OS = overall survival.

The relationship between the clinical data of 2 distinct level groups of GNPDA1, including gender, age, grading, and tumor TNM staging, was analyzed through data analysis, as depicted in the heatmap (Fig. 4A). Interestingly, the K–M curve demonstrated that patients with high expression of GNPDA1 had a lower OS rate in HNSCC cases (Fig. 4B). Moreover, the receiver operating characteristic curve indicated that GNPDA1 could potentially serve as a predictive factor for 1, 3, and 5-year survival rate (Fig. 4C). Furthermore, gender appeared to play a significant role in the expression of GNPDA1, with male patients displaying notably higher levels compared to female patients (Fig. 4D). The expression of GNPDA1 was also found to be closely linked to tumor grading, with G stage patients exhibiting significantly elevated expression levels (Fig. 4E). Notably, GNPDA1 expression showed a direct correlation with staging, as patients in the N stage displayed significantly higher levels of GNPDA1 compared to those in the N0 stage (Fig. 4F). Additionally, the expression of GNPDA1 was associated with the T phase, with higher levels observed in patients with more advanced T stages than those with lower T phases (Fig. 4G). These findings collectively suggest that GNPDA1 expression is intricately linked to tumor progression, highlighting its potential as a prognostic marker in clinical settings.^[21]^

### 3.4. GNPDA1 and ER-related genes regulate HNSCC, demonstrating significant impact on cancer development and progression

**Figure 5.**
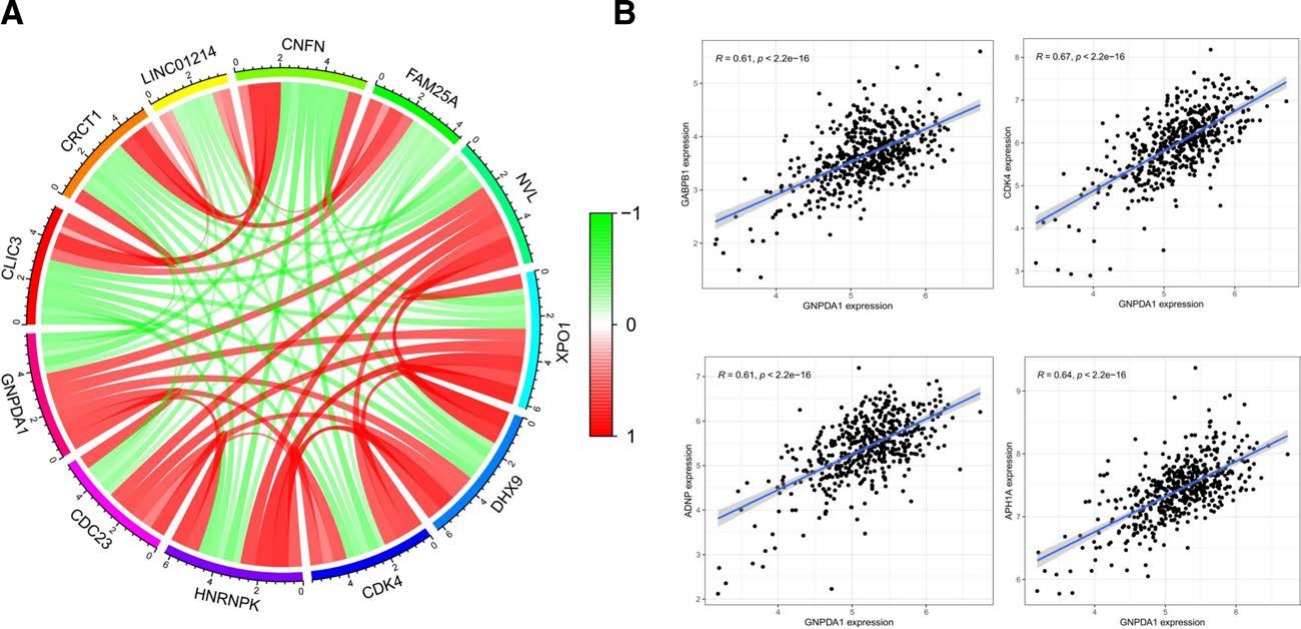
Co-expressed genes of GNPDA1 in HNSCC. (A) Based on the Pearson’s correlation coefficient, the circular graph shows the top 12 genes in the HNSC-TCGA cohort. (B) Four genes in the HNSC-TCGA database had Pearson coefficients > 0.6, *P* < .001. GNPDA1 = glucosamine-6-phosphate isomerase 1, HNSCC = head and neck squamous cell carcinoma, HNSC-TCGA = head and neck squamous cell carcinoma-the Cancer Genome Atlas.

Analysis of HNSCC data revealed 75 genes that exhibited co-expression with GNPDA1. These genes, with a Pearson coefficient > 0.6 and a *P*-value of <.001, were found to directly or indirectly influence the expression of GNPDA1. Notably, GNPDA1 also emerged as a key regulator of these associated genes. The correlation analysis rendered a circular graph showcasing the top 12 genes that displayed positive or negative correlation with GNPDA1 expression (Fig. 5A). Furthermore, an integration of data from multiple databases highlighted GABPB1, CDK4, ADNP, and APH1A as genes significantly linked to GNPDA1 expression in HNSCC (Fig. 5B). This discovery suggests a pivotal regulatory role of the proteins encoded by these 4 genes in modulating GNPDA1 expression in patients with HNSCC.

### 3.5. GNPDA1 plays a crucial role in controlling the aggressive behavior and immune evasion of tumor cells in HNSCC

**Figure 6.**
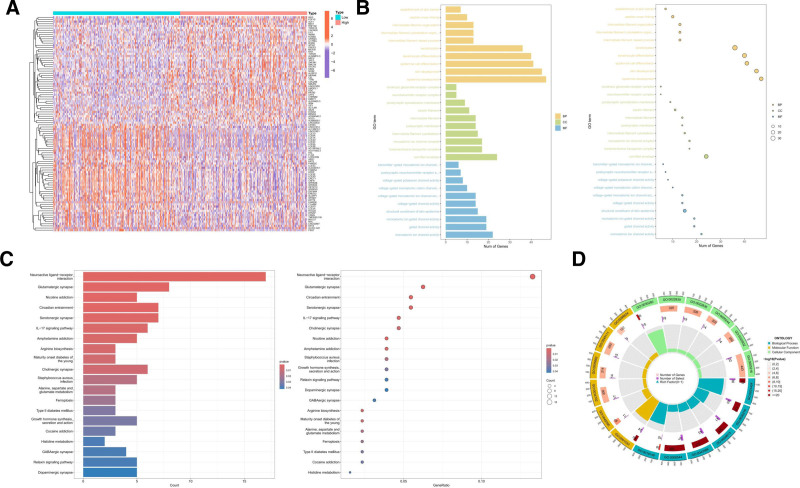
Functional enrichment analysis of GNPDA1 in HNSCC. (A) The heatmap displays genes with high and low expression of GNPDA1. (B) Bar and bubble plots depicting the enrichment pathways obtained from the GO analysis of DEGs. (C) Bar chart and bubble plot of enrichment pathways from KEGG analysis. (D) GO enrichment analysis analyzed the regulatory pathways of DEGs enrichment. DEGs = differentially expressed genes, GO = gene ontology, GNPDA1 = glucosamine-6-phosphate isomerase 1, HNSCC = head and neck squamous cell carcinoma, KEGG = Kyoto encyclopedia of genes and genomes.

A comprehensive analysis was conducted to compare the gene expression levels associated with GNPDA1 in both high and low expression states. A total of 548 DEGs were identified, with 395 genes showing upregulation and 153 genes showing downregulation. The heatmap visually represents the top 50 upregulated genes and the top 50 downregulated genes (Fig. 6A). GO analysis shows that DEGs are mainly enriched in immune related pathways (Fig. 6B). Additionally, Kyoto encyclopedia of genes and genomes analysis highlighted pathways such as extracellular matrix receptor, tight junctions, and cell adhesion molecules interactions (Fig. 6C). Moreover, GO enrichment analysis revealed that in the case of low expression of GNPDA1, pathways related to immunoglobulin complexes and T cell receptor complexes were significantly enriched (Fig. 6D). These findings collectively suggest a potential role of GNPDA1 in the migration ability of HNSCC cells.

### 3.6. GNPDA1 significantly reduces immune cell infiltration in the TME of HNSCC

**Figure 7.**
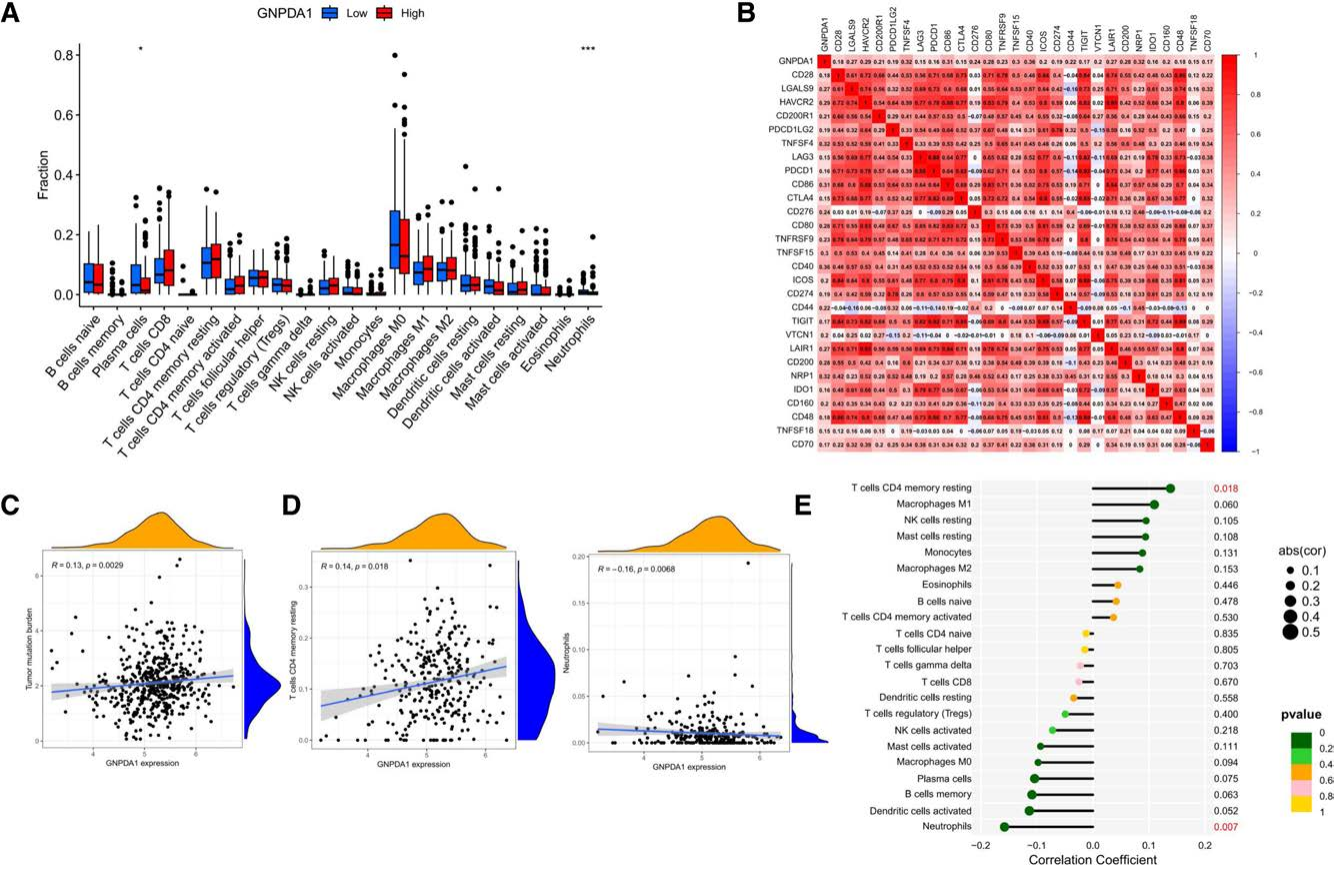
GNPDA1 affects immune cell infiltration in HNSCC. (A) Low and high expression of GNPDA1 in 24 immune cells infiltrates of HNSCC patients. (B) GNPDA1 expression and ICP in HNSCC. (C) GNPDA1 expression in HNSCC and TMB. (D) GNPDA1 expression and CD4-Tt and neutrophil infiltration. (E) GNPDA1 expression and infiltration of 22 immune cells in HNSCC. (*P < .05; **P < .01; ***P < .001). GNPDA1 = glucosamine-6-phosphate isomerase 1, HNSCC = head and neck squamous cell carcinoma, ICP = immune checkpoint, TMB = tumor mutation burden.

In the analysis depicted in the figure, the differences in immune cell infiltration levels between various cell types such as plasma cells, Treg cells, macrophages M0, M2, DC cells, and activated mast cells based on high and low GNPDA1 expression were observed. It was found that the group with high GNPDA1 expression showcased a significant reduction in immune cell infiltration compared to the low expression group (Fig. 7A). However, the analysis of TMB indicated that the group with high GNPDA1 expression exhibited obviously higher levels than the low expression group (Fig. 7C), suggesting that patients with high GNPDA1 protein expression displayed enhanced immune responses. We conducted a correlation analysis between GNPDA1 and 28 well-known ICP genes, revealing a positive correlation with most ICP genes (Fig. 7B). Moreover, the relationship between GNPDA1 and immune cell infiltration alongside 22 immune cells was further investigated. The outcomes elucidated a positive correlation between GNPDA1 expression and decreased activation of NK cells, neutrophils, activated mast cells, macrophages M0, plasma cells, activated DC cells, CD8^+^T cells, memory B cells, Treg cells, and naive CD4^+^T cells. Conversely, a positive correlation was observed between GNPDA1 expression and increased infiltration of quiescent CD4^+^T cells, macrophages M1, M2, quiescent NK cells, monocytes, eosinophils, and naive B cells (Fig. 7D, E).

### 3.7. GNPDA1 gene influences drug response in HNSCC patients, impacting treatment effectiveness significantly

**Figure 8.**
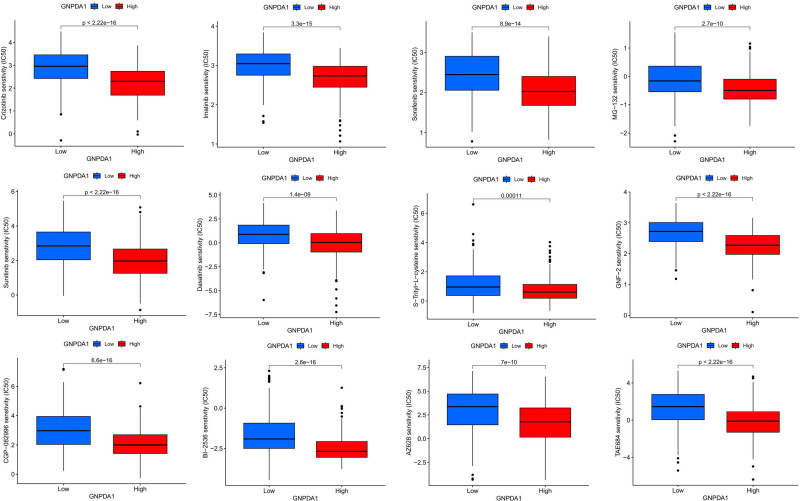
Correlation between GNDPA1 expression and chemosensitivity. GNPDA1 = glucosamine-6-phosphate isomerase 1.

Patients with high GNPDA1 expression levels showed a significant improvement in drug sensitivity to various treatments including AZ628, BI-2536, CGP-082996, Crizotinib, Dasatinib, Erlotinib, GNF-2, Imatinib, MG-132, Paclitaxel, Rapamycin, Sorafenib, S-Trityl-cystfine, Sunitinib, and TAE684. These findings suggest that GNPDA1 may play a crucial role in enhancing the effectiveness of these drugs (Fig. 8).

## 4. Discussion

Studies have demonstrated the significant impact of pembrolizumab, a humanized anti-PD-1 monoclonal antibody, on boosting antitumor immune response in the treatment of HNSCC.^[5]^ Despite its breakthrough potential, the effectiveness of pembrolizumab varies among patients due to differing tumor microenvironments (TME).^[8]^ This limitation highlights the critical need for identifying novel biomarkers and immune targets to enhance treatment outcomes for a broader population of HNSCC patients. By exploring alternative avenues, researchers aim to optimize the therapeutic potential of immunotherapy and improve overall patient responses within this challenging cancer landscape.

GNPDA1 is an enzyme that plays a critical role in cellular metabolism by facilitating the conversion of d-6-glucosamine phosphate to fructose-6-phosphate and ammonium salts.^[22]^ This process is essential for the synthesis of fructose-6-phosphate, which is crucial for transferring hexosamine from structural macromolecules to glycolytic pathways. The high metabolic rate of GNPDA1 in vivo suggests its potential impact on cellular energy metabolism.^[23]^ The reprogramming of metabolism is a key characteristic of tumor cells, allowing them to effectively balance their energy requirements, anabolism, and glycolysis to support rapid proliferation.^[24,25]^ In the case of HNSCC, various metabolic pathways, including aerobic glycolysis, pentose phosphate, the tricarboxylic acid cycle, fatty acids, and glutathione pathways, are implicated in the disease progression.^[26,27]^ The aberrant expression of metabolism-related genes in HNSCC is linked to poor patient prognosis, indicating the significance of targeting metabolic reprogramming for therapeutic interventions. While GNPDA1 has been detected in certain cancer cell lines, its specific role in tumorigenesis remains unclear. It is hypothesized that GNPDA1 may provide additional energy to HNSCC cells by linking the hexosamine system and glycolytic pathway, thereby supporting the synthesis of biological macromolecules and facilitating rapid cell proliferation. Understanding the precise role of GNPDA1 in the context of HNSCC metabolism could pave the way for novel therapeutic strategies targeting metabolic pathways to effectively combat the disease.

In our study, we delved into the analysis of GNPDA1 transcriptome data across 23 different types of tumors. The findings revealed a consistent trend of elevated GNPDA1 expression levels across almost all tumor types. Particularly noteworthy was the significant upregulation of GNPDA1 expression in HNSCC when compared to normal tissues, both in paired and unpaired samples. Solid tumors are formed by the complex fusion of stromal cells, malignant cells, and immune cells, providing a unique microenvironment for GNPDA1 activity.^[28]^ To better comprehend the role of GNPDA1 in solid tumors, it is essential to ascertain its precise localization and distribution within these tumors. Our comprehensive single-cell analysis unveiled revealed GNPDA1 is mainly at an increased level in malignant cells, while exhibiting widespread expression in many immune cells. Studies indicate that GNPDA1 may impact the immune infiltration of immune cells in cancer patients, hinting at its involvement in HNSCC progression beyond just malignant cells.^[28,29]^ Therefore, further investigation into the role of GNPDA1 in immune cells within solid tumors is imperative for a comprehensive understanding of its implications.

The study examined the importance of GNPDA1 in predicting outcomes for patients with HNSCC and its clinical implications. The research revealed that increased levels of GNPDA1 were linked to poorer OS rates and more advanced disease progression in HNSCC patients. Analysis of clinical data further demonstrated a strong association between GNPDA1 expression levels and the G and N stages of HNSCC, with elevated GNPDA1 expression observed in patients with more advanced disease stages. In addition, analyzing GNPDA1 is an important factor affecting the prognosis of HNSCC patients in terms of age and grading. The study also explored the relationship between GNPDA1 and other genes in HNSCC, revealing abnormal expression patterns in genes that interact with GNPDA1, forming a regulating network that influences the development and progression of the disease. By analyzing interactions with the GNPDA1 protein using the STRING database, researchers identified 4 key genes (GABPB1, CDK4, ADNP, and APH1A) that play important roles in cellular processes related to cancer. GABPB1 is involved in mitochondrial biosynthesis and activates GNPDA1 expression,^[30]^ while CDK4 regulates endoplasmic reticulum (ER) stress and cellular functions.^[31]^ Additionally, ADNP is crucial for gene activation and promotes a specific type of immune response,^[32]^ and APH1A is associated with ER and oxidative stress responses.^[33]^ These findings suggest that the activation of GNPDA1 may influence the ER stress response in HNSCC and that the identified genes collaborate to enhance tumor cell survival in adverse conditions. Understanding these interactions could provide insights into potential therapeutic targets for improving outcomes in patients with HNSCC.

Understanding the role of GNPDA1 and its associated activation pathways is crucial in unraveling its impact on tumor regulation. Through gene set enrichment analysis, we delved deeper into the functions of GNPDA1, revealing its involvement in the regulation of malignant phenotypes in HNSCC. Notably, GNPDA1 also participates in pathways related to extracellular matrix receptor, tight junctions and cell adhesion molecules interactions, all crucial for the invasion and migration ability of tumor cells.^[34]^ Our comprehensive analysis through GO and gene set enrichment analysis uncovered the extensive influence of GNPDA1 on immune-related pathways, shedding light on its potential to shape the TME within tumors. By examining the immune cell content in the HNSCC TME, we observed a significant impact of GNPDA1 expression on immune cell infiltration within solid tumors. Interestingly, high GNPDA1 expression levels were associated with reduced infiltration of various immune cells, including NK cells, neutrophils, mast cells, macrophages, plasma, DC, Treg, CD8^+^T and initial CD4^+^T cells. These findings not only corroborate our single-cell analysis but also highlight the critical role of CD4^+^T and CD8^+^T cells in the HNSCC TME. NK cells play a key role in recognizing and eliminating target cells affected by various conditions, including cancer.^[35]^ The regulatory function of GNPDA1 in solid tumors extends to inhibiting immune cell infiltration, leading to weaker immune responses and, consequently, poorer prognoses for patients with high GNPDA1 expression levels. In conclusion, GNPDA1 emerges as a key player in solid tumor development, exerting its influence on both tumor progression and immune cell function in the TME. Its ability to modulate immune responses underscores its significance as a potential therapeutic target for enhancing antitumor immune responses and improving patient outcomes.

Cancer cells have the ability to evade the immune system by expressing ICP antigens. PD-1/PD-L1, CTLA-4 inhibitors have immunological inhibition effects on ICP genes and have been widely used in clinical practice.^[36]^ Despite their use, the effectiveness of these inhibitors varies among individuals. Research findings point to a strong correlation between GNPDA1 expression and multiple ICP genes, notably CD276, which is a key immunosuppressive gene associated with poor prognosis in HNSCC. CD276 plays a crucial role in allowing HNSCC stem cells to evade immune surveillance, impacting the function of CD8^+^ T cells in cancer stem cells.^[37,38]^ The connection between GNPDA1 and these ICP genes suggests its involvement in mediating immune evasion in HNSCC. Future studies are expected to delve deeper into understanding the relationship between GNPDA1 and ICP genes, uncovering the specific mechanisms through which they regulate each other. This research paves the way for potential advancements in cancer treatment strategies targeting immune evasion mechanisms.

Our research has highlighted the potential effectiveness of targeting GNPDA1 as a therapeutic strategy for treating HNSCC. GNPDA1 plays a crucial role in shaping the immune environment within tumor cells and has a significant impact on the prognosis of HNSCC patients. Despite these promising findings, there are still some challenges that need to be addressed. In the future, a series of basic experiments can be conducted to investigate the specific mechanism by which GNPDA1 affects immune cells in TME. Furthermore, the specific role of GNPDA1 prognostic indicator for HNSCC must be validated in clinical settings. Nevertheless, our study provides valuable insights for future research initiatives aiming to identify novel targeted therapies for HNSCC.

## 5. Conclusion

The study examined the significance of GNPDA1 in predicting the prognosis of HNSCC. The findings indicate that GNPDA1 serves as a potential biomarker for forecasting the outcome of HNSCC. It is closely linked to immune evasion and immune cell infiltration in this type of cancer. This highlights the potential of GNPDA1 as a valuable tool in predicting HNSCC prognosis.

## Author contributions

**Visualization:** Dunhui Yang.

**Writing – original draft:** Peng Liu.

**Writing – review & editing:** Peng Liu, Ruixia Ma.
